# *Chlamydia pneumoniae* effector chlamydial outer protein N sequesters fructose bisphosphate aldolase A, providing a benefit to bacterial growth

**DOI:** 10.1186/s12866-014-0330-3

**Published:** 2014-12-21

**Authors:** Kasumi Ishida, Junji Matsuo, Yoshimasa Yamamoto, Hiroyuki Yamaguchi

**Affiliations:** Department of Medical Laboratory Science, Faculty of Health Sciences, Hokkaido University, Sapporo, Hokkaido 060-0812 Japan; Research Fellow of Japan Society for the Promotion of Science, Tokyo, 102-0083 Japan; Department of Biomedical Informatics, Osaka University Graduate School of Medicine, Suita, Osaka 565-0871 Japan; Japan Science and Technology Agency/Japan International Cooperation Agency, Science and Technology Research Partnership for Sustainable Development (JST/JICA, SATREPS), Osaka, Japan; Osaka Prefectural Institute of Public Health, Higashinari-ku, Osaka, 537-0025 Japan

**Keywords:** Chlamydia pneumoniae, Type III secretion, Effectors, Chlamydial outer protein N

## Abstract

**Background:**

Pathogenic chlamydiae are obligate intracellular pathogens and have adapted successfully to human cells, causing sexually transmitted diseases or pneumonia. Chlamydial outer protein N (CopN) is likely a critical effector protein secreted by the type III secretion system in chlamydiae, which manipulates host cells. However, the mechanisms of its action remain to be clarified. In this work, we aimed to identify previously unidentified CopN effector target in host cells.

**Results:**

We first performed a pull-down assay with recombinant glutathione S-transferase (GST) fusion CopN proteins (GST–CpCopN: *Chlamydia pneumoniae* TW183, GST–CtCopN: *Chlamydia trachomatis* D/UW-3/CX) as “bait” and soluble lysates obtained from human immortal epithelial HEp-2 cells as “prey”, followed by SDS-PAGE with mass spectroscopy (MS). We found that a host cell protein specifically bound to GST–CpCopN, but not GST–CtCopN. MS revealed the host protein to be fructose bisphosphate aldolase A (aldolase A), which plays a key role in glycolytic metabolism. We also confirmed the role of aldolase A in chlamydia-infected HEp-2 cells by using two distinct experiments for gene knockdown with an siRNA specific to aldolase A transcripts, and for assessment of glycolytic enzyme gene expression levels. As a result, both the numbers of chlamydial inclusion-forming units and RpoD transcripts were increased in the chlamydia-infected aldolase A knockdown cells, as compared with the wild-type HEp-2 cells. Meanwhile, chlamydial infection tended to enhance expression of aldolase A.

**Conclusions:**

We discovered that one of the *C. pneumoniae* CopN targets is the glycolytic enzyme aldolase A. Sequestering aldolase A may be beneficial to bacterial growth in infected host cells.

## Background

Obligate intracellular pathogens *Chlamydia pneumoniae* and *Chlamydia trachomatis* are major clinical and public health concerns, and cause pneumonia [1,2] and the most prevalent sexually transmitted diseases worldwide [3,4], respectively. Although *C. pneumoniae* is also associated with chronic inflammatory diseases such as asthma [5] and atherosclerosis [2], its etiological role remains to be clarified.

Chlamydiae undergo a unique biphasic developmental cycle, with a morphological change between the elemental body (EB), which is an infectious form, and reticulate body (RB), which is a replicative form, into the surrounding plasma membrane, the so-called inclusion membrane [6-8]. To complete their developmental cycle, chlamydiae utilize the type III secretion system (T3SS) to translocate several effector proteins across the inclusion membrane [9]. Accumulating evidence from experiments with *Shigella* or *Salmonella* have shown that T3SS effectors play a critical role in bacterial invasion and maturation, and maintenance of host cell viability, which is directly related to bacterial pathogenesis [10,11]. Although several chlamydial T3SS effectors such as translocated actin recruiting phosphoprotein (TARP) [12], inclusion membrane protein A (IncA) [13], and chlamydial outer protein N (CopN) [14] have been documented, mechanisms of host cell manipulation evoked by these effectors remain to be clarified.

Gram-negative bacterial T3SS is a complex of multiple proteins including tip, needle, rod and plug proteins, and ATPase [15], and the effector proteins translocating across the plasma membrane are strictly regulated by the plug proteins. For instance, MxiC, a plug protein of *Shigella flexneri*, regulates effector secretion [16,17]. The activation of *Yersinia* T3SS effector secretion is controlled by YopN/TyeA as a plug complex of *Yersinia* T3SS in response to extracellular calcium and/or contact with eukaryotic cells [18,19]. Also, translocation of some plug proteins such as YopN or MxiC into eukaryotic host cells has been documented [16,20]. Thus, these bacterial plug molecules, possibly with effector function, have a central role into controlling T3SS effector secretion.

Recent work has shown that chlamydial CopN, which is homologous to YopN and MxiC but with low similarity, is a T3SS plug protein, and is secreted by a *Yersinia* type III apparatus [14,21,22]. Also, CopN domain structure analysis using gel filtration assay with His-tagged recombinant proteins has revealed that CopN binds to tubulin, which prevents microtubule assembly [23]. Furthermore, yeast two-hybrid analysis has shown the essential role of the CopN effector protein in *C. pneumoniae* intracellular growth [24]. Thus, these findings support that chlamydial CopN has a critical role as a T3SS plug with effector function in the chlamydial host manipulation system. Whether the CopN targets tubulin alone remains to be determined.

Here, we showed that *C. pneumoniae* CopN interacted with a glycolytic enzyme, fructose bisphosphate aldolase A (aldolase A). Our data also suggested that sequestering aldolase A was beneficial to bacterial growth in infected cells.

## Methods

### Human cell lines

The immortal human epithelial cell lines HEp-2 and HeLa were purchased from American Type Culture Collection (Manassas, VA, USA) and Riken Cell Bank (Tsukuba, Japan), respectively. Both the cell lines were cultured at 37°C in 5% CO_2_ in Dulbecco’s Modified Eagle’s Medium (DMEM; Sigma, St. Louis, MO, USA) containing 10% heat-inactivated fetal calf serum (FCS), and antibiotics [10-μg/ml gentamicin, 10-μg/ml vancomycin and 0.5-μg/ml amphotericin B (Sigma)] (GVA antibiotics) [25,26].

### Chlamydiae and growth conditions

*C. pneumoniae* TW183 strain was kindly provided by G. Byrne, University of Tennessee (Memphis). *C. trachomatis* D/UW3 CX strain (VR-885) was purchased from ATCC. Chlamydiae were propagated in HEp-2 and HeLa cells, as described previously [25,26]. DMEM supplemented with 20% FCS and GVA antibiotics was used for cell culture. The infected HeLa and HEp-2 cells were harvested at 48 and 72 h after infection, respectively, and stored at −80°C. The cell suspensions were disrupted by freezing–thawing and centrifugation at 400 × *g* to remove cell debris. The bacteria were collected by centrifugation at 1000 × *g* and resuspended in sucrose–phosphate–glutamic acid buffer (0.2-M sucrose, 3.8-mM KH_2_PO_4_, 6.7-mM Na_2_HPO_4_ and 5-mM l-glutamic acid). The bacteria were stored at −80°C until use. The number of bacterial infectious particles equal to EBs was determined by inclusion-forming unit (IFU) assay [27] as described below. Both bacterial genomic DNAs were extracted with High Pure PCR Template Preparation Kit (Roche, Indianapolis, IN, USA) and used as a template DNA for *copN* genes cloning.

### Construction of recombinant CopNs

Full-length *copN* genes [*CpcopN*: *C. pneumoniae* TW183, CpB0334 (NC_005043.1); *CtcopN*: *C. trachomatis* UW-3/CX serovar D, CT_089 (NC_000117.1)] were amplified from each of the genomic DNA templates. The amplified DNA products were cloned to the downstream of glutathione S-transferase (GST) gene region into pGEX-6P-1 plasmid (GE Healthcare Bio-Sciences AB, Uppsala, Sweden). pGEX-6P-1 plasmid was kindly provided by I. Hirai, University of the Ryukyus (Okinawa, Japan). GST-fused recombinant proteins were finally expressed in *Escherichia coli* BL21 (DE3) in Luria–Bertani medium with 0.1-mM isopropyl-1-thio-β-d-galactopyranoside for 4 h at 37°C. The bacteria were collected by centrifugation and then lysed chemically using bacterial protein extraction reagent (Thermo Fisher Scientific, Waltham, MA, USA). After centrifugation to remove bacterial debris, GST-fusion proteins were prepared from the supernatant by using glutathione–agarose beads (Thermo Fisher Scientific) and the protein concentrations were determined using the Bradford assay. The solutions were adequately dispensed and stored at −80°C until use.

### Pull-down assay with mass spectrometry (MS)

GST-fusion proteins were mixed with the soluble fraction obtained from HEp-2 cells, and then incubated for 4–12 h at 4°C in cell lysis buffer containing 40-mM Tris–HCl (pH 7.5), 150-mM sodium chloride, 0.5% Triton-X-100, and 1% protease inhibitor cocktail (Nacalai Tesque, Kyoto, Japan) in the presence of glutathione–agarose beads (Thermo Fisher Scientific). After washing with 50-mM Tris–HCl (pH 6.8), the captured proteins on beads were eluted with sample buffer for SDS-PAGE. Protein profiles into the eluted solutions were visualized by SDS-PAGE with Coomassie brilliant blue staining, and the specific band presumably captured by CopN was cut out from the gel, and then used for nano-liquid chromatography tandem MS (nano-LC-MS/MS) analysis (Japan Bio Services, Saitama, Japan). The MS analysis was carried out by the Japan Bio Services.

### RNA extraction and conventional or quantitative reverse-transcription polymerase chain reaction (qRT-PCR)

Total RNA was extracted from chlamydia-infected or uninfected HEp-2 cells using High Pure RNA Isolation Kit (Roche) according to the manufacturer’s protocol. Reverse transcription of 500-ng total RNA was performed with ReverTraAce qPCR RT Master Mix (Toyobo, Osaka, Japan). Resultant cDNAs were used for PCR amplification with the following primers: (*gapdh*) (forward 5′-AAC GGG AAG CTC ACT GGC ATG-3′; reverse 5′-TCC ACC ACC CTG TTG CTG TAG-3′) [28], *aldolase A* (forward 5′-CGC AGA AGG GGT CCT GGT GA-3′; reverse 5′-CAG CTC CTT CTT CTG CTC CGG GGT-3′) [29], *enolase* (forward 5′-GAG CTC CGG GAC AAT GAT AA-3′; reverse 5′-CTG TTC CA TCC ATC TCG ATC-3′) [30], *β-actin* (forward 5′-GAC CAC ACC TTC TAC AAT GAG-3′; reverse 5′-GCA TAC CCC TCG TAG ATG GG-3′) [31] and chlamydial *16S rRNA* (forward 5′-CGG CGT GGA TGA GGC AT-3′; reverse 5′-TCA GTC CCA GTG TTG GC-3′) [32]. PCR conditions were 95°C for 10 min, followed by 25–40 cycles of 95°C for 30 s, 55 or 60°C for 30 s and 72°C for 45 s, followed by 72°C for 10 min. PCR products were visualized on 2% agarose gels with ethidium bromide. For qRT-PCR, resultant cDNAs were also amplified by SYBR Green Realtime PCR Master Mix (Toyobo) using the above primer sets. The transcript amount of target gene products was expressed as a ratio to that of *β-actin*.

### Establishment of aldolase A knockdown cells and chlamydial infection

Transient aldolase A knockdown HEp-2 cells were established by 24-h transfection of cells with siRNA as follows: passenger strand, 5′-CCG AGA ACA CCG AGG AGA A dTdT-3′; guide strand, 5′-UUC UCC UCG GUG UUC UCG G dTdT-3′. Non-targeting scramble RNA was also constructed by Hokkaido System Science (Sapporo, Japan) as follows: passenger strand, 5′-UUC UCC GAA CGU GUC ACG UdTdT-3′; guide strand, 5′-ACG UGA CAC GUU CGG AGG AGA AdTdT-3′. Transfection of siRNA (or scramble RNA) to cells was performed with a transfection reagent, Multifectam (Promega, Madison, WI, USA), according to the manufacturer’s protocol as below. Transfection complexes were prepared by incubating 20 μL of the prepared siRNA [or scramble RNA (1.0 pmol/μl)] with 10-μl Multifectam for 30 min at room temperature. After incubation, total transfection complexes were added to HEp-2 cells adjusted to 1 × 10^5^ cells, and then incubated for 24 h at 37°C in 5% CO_2_. Expression of aldolase A was confirmed by Western blotting. At 24 h after transfection, HEp-2 cells were infected with chlamydiae at MOI 5 by centrifugation at 400 × *g* for 1 h. After washing uninfected bacteria with DMEM, chlamydia-infected cells were cultured for up to 72 h, and bacterial growth was monitored by IFU assay and Western blotting.

### IFU assay and inclusion images

The number of chlamydial infectious particles (EBs) was determined by IFU assay by counting chlamydial inclusions formed in HEp-2 cells using fluorescein-isothiocyanate-conjugated monoclonal anti-*Chlamydia* antibody specific for *Chlamydia* lipopolysaccharide (with Evans Blue) (Denka Seiken, Tokyo, Japan), as described previously [27]. In some experiments, the cells were grown on cover glass, and chlamydial inclusion images were captured using a digital camera with a fluorescence microscope.

### Western blotting

Infected and uninfected cells collected from each culture were boiled for 5 min in SDS sample buffer containing 2-mercaptoethanol. After boiling, each sample was sonicated to remove viscosity. Samples were loaded and separated by 10% (w/v) SDS-PAGE. Separated proteins were transferred to polyvinylidene difluoride membranes by semi-dry electroblotting. The membrane was blocked with 5% skimmed milk in Tris-buffered saline including Tween-20 (TBST). After washing the blocked membrane with TBST, the membrane was incubated with a primary antibody [mouse anti-GST monoclonal antibody (Nacalai Tesque), rabbit anti-human aldolase A polyclonal antibody (Cell Signaling Technology, Danvers, MA, USA), mouse anti-α-tubulin monoclonal antibody (Sigma–Aldrich) and rabbit anti-RpoD polyclonal antibody (prepared by Sigma–Aldrich)] for 1 h at room temperature. This was followed by a secondary antibody [horseradish peroxidase (HRP)-conjugated goat anti-mouse IgG and IgM antibody (Jackson ImmunoResearch, West Grove, PA, USA) and HRP-conjugated goat anti-rabbit IgG antibody (KPL, Gaithersburg, MD, USA)] for 1 h at room temperature. Labeled proteins were visualized with Pierce Western Blotting Substrate (Thermo Fisher Scientific).

### Statistical analysis

Data were expressed as mean ± standard deviation (SD). Comparison of data was performed using an unpaired Student *t* test. A value of *p* < 0.05 was considered significant. All experiments were repeated at least three times.

## Results and discussion

Chlamydiae are obligate intracellular pathogens infecting various vertebrate cells, including human. To optimize the intracellular niche for their propagation, chlamydiae utilize several T3SS effector proteins. Several chlamydial T3SS effectors including TARP [12], IncA [13] and CopN [14] have been reported with detailed protein structures, and these provide hints for elucidating how chlamydiae manipulate host cells, resulting in successful adaptation. CopN effector is well conserved among chlamydiae, functioning both as the T3SS plug and a secreted effector protein [23]. The effector protein is responsible for a central role in chlamydial adaptation to host cells. Recent studies have elegantly demonstrated that CopN manipulates host cells, causing mitotic arrest [33], and that CopN interacts with tubulin, preventing microtubule assembly [23,24]. However, whether the CopN targets tubulin alone remains undetermined. In this work, we therefore attempted to find previously unidentified chlamydial CopN effector targets in host cells.

### *C. pneumoniae* CopN interacts with human aldolase A

**Figure 1:**
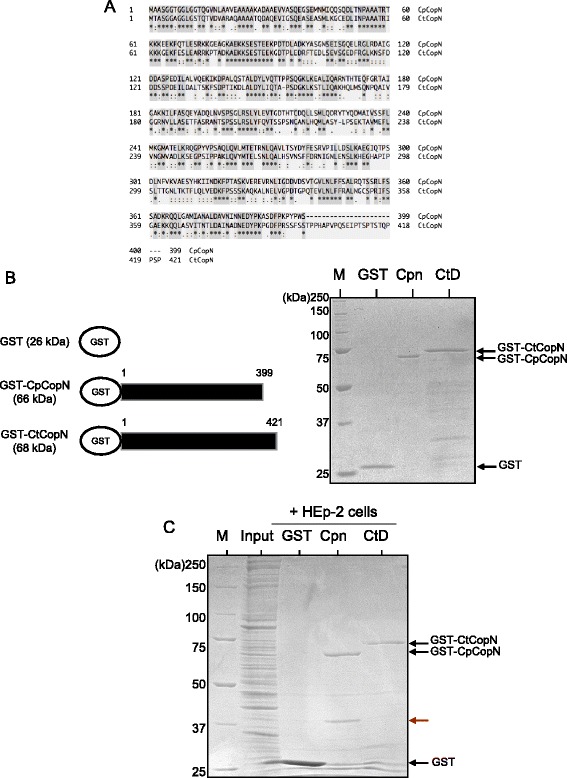
**Sequence similarity between** ***C. pneumoniae*** **and** ***C. trachomatis*** **CopNs and representative pull-down images. (A)** Sequence similarity between *C. pneumoniae* and *C. trachomatis* CopNs. Amino acid sequence data were obtained from NCBI genome site (http://www.ncbi.nlm.nih.gov/genome/). *C. pneumoniae* TW183 CopN, CpB0334 (accession number: NC_005043.1). *C. trachomatis* UW-3/CX serovar D CopN, CT_089 (accession number: NC_000117.1). Alignment was constructed by web software, UniProt (http://www.uniprot.org/). *C. pneumoniae* CopN had 48% homology as compared with *C. trachomatis* CopN. Stars highlighted gray indicate identical amino acids. **(B)** Protein expression profiles of constructed recombinant GST-fused CopNs. Cpn, *C. pneumoniae* CopN full length (399 aa) (GST-CpCopN). CtD, *C. trachomatis* CopN full length (421 aa) (GST-CtCopN). GST, GST alone. **(C)** Representative pull-down images with GST-fused chlamydial CopNs in HEp-2 cell lysates. Red arrow shows a specific band captured by GST–CpCopN, but not GST–CtCopN, in the HEp-2 cell lysates.

**Figure 2:**
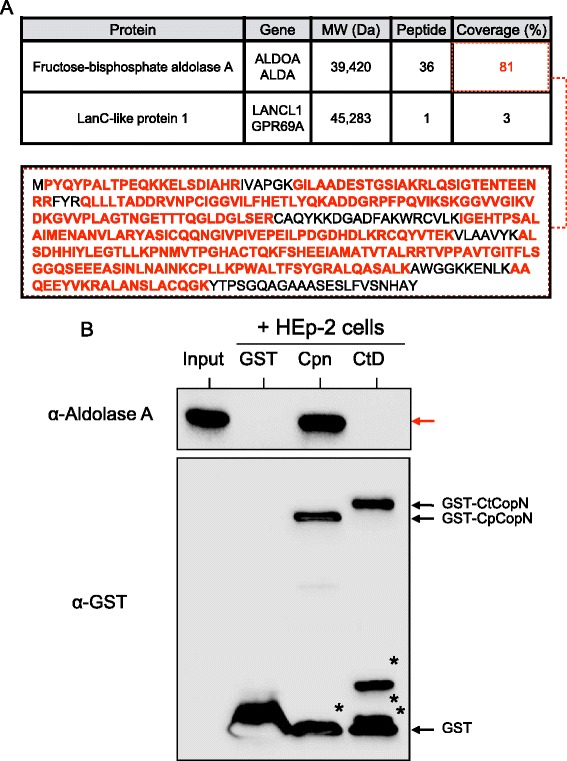
**Pull-down assay with MS analysis and Western blotting showing that** ***C. pneumoniae*** **CopN interacts with human aldolase A. (A)** MS data with nano-LC-MS/MS. Bold red amino acid sequences, peptide matched sequences. **(B)** Representative Western blotting pattern with a specific aldolase A antibody for elution obtained from pull-down assay with GST-fused chlamydial CopNs and HEp-2 cell lysates. Input, HEp- 2 cell lysates. GST, GST alone. Cpn, GST-fused *C. pneumoniae* CopN (GST-CpCopN). CtD, GST-fused *C. trachomatis* CopN (GST-CtCopN). Red arrow, aldolase A. Asterisk, fragmented proteins.

The CopN protein family, which functions as T3SS plug proteins with effector functions, is strictly conserved with weak sequence homology to chlamydiae and other Gram-negative bacteria [9], and the sequence similarity of CopNs used for this study was 48% (Figure 1A). To identify the host proteins that interact with chlamydial CopN, we performed a pull-down assay with GST-fused recombinant CopN proteins (*C. pneumoniae*: GST–CpCopN, *C. trachomatis:* GST–CtCopN) and HEp-2 cell lysates. Coomassie brilliant blue staining revealed that unrelated contaminants in the recombinant fractions were minimal in purified GST-fused protein fractions (Figure 1B). As a result, a specific band with ~40-kDa molecular size was detected in the eluted pull-down fraction with GST–CpCopN, but not in that with GST–Ctr CopN or GST alone (Figure 1C, arrowhead). It was a possible candidate for CopN target molecule, thus, the band cut out from the gel was analyzed by nano-LC/MS/MS. As a result, MS revealed two distinct eukaryotic proteins to be aldolase A, which is involved in glycolytic metabolism, and lancC-like protein, which is a lanthionine synthetase component (Figure 2A). The peptide hit numbers and coverage against aldolase A were significantly high compared with those for lancC-like protein (Figure 2A); therefore, we concluded that GST–CpCopN target protein might be a glycolytic enzyme, aldolase A. To confirm this, we performed Western blotting with a specific antibody against human aldolase A in pull-down eluted fractions. As expected, Western blotting clearly indicated a specific band reacting with the specific antibody, demonstrating that the GST-fused CpCopN target molecule was aldolase A, which is an important enzyme in glycolysis and glyconeogenesis, and affects actin polymerization via the Wiskott–Aldrich syndrome protein (WASP)/Arp2/3 pathway [34] (Figure 2B).

To the best of our knowledge, we are the first to show that chlamydial CopN could capture human aldolase A. Intriguingly, because no specific protein was seen in the case of *C. trachomatis* CopN, this capturing was specific to *C. pneumoniae* CopN effector. The exact reason for specific binding remains unclear, but it is likely associated with the low amino acid sequence similarity (48%) between the CopNs. Furthermore, previous work has indicated that while *C. pneumoniae* directly secretes CopN into the host cytosol across the inclusion membrane, *C. trachomatis* CopN is embedded in the inclusion membrane, and is never translocated to the host cell cytosol, suggesting that the target molecule of *C. trachomatis* CopN may be buried in the membrane fraction, without high solubility [14]. Taken together, the findings imply a possible different effector target and function between *C. pneumoniae* and *C. trachomatis* CopNs.

Our pull-down system with GST-fused CopN proteins and cell lysates unfortunately could not capture microtubulin, which is a cytoskeletal fiber, as described previously [23]. That research group used a gel filtration assay with microtubulin pelleting required for large amounts of recombinant proteins [23]; thus, there might have been a critical difference in detection sensitivity. Alternatively, the previous study also indicated that, in contrast to tubulin, *C. pneumoniae* CopN could not bind directly to microtubulin [23]; therefore, a possible explanation may be in our pull-down system with whole cell lysates, which naturally conserves cellular molecular structures.

### Chlamydial growth is stimulated in aldolase A knockdown cells

**Figure 3:**
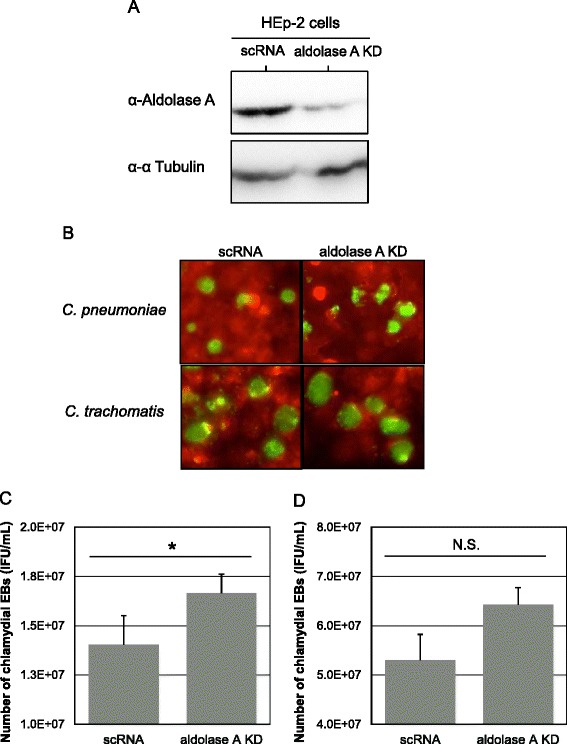
**Changes in chlamydial infectious particle numbers in aldolase A knockdown cells. (A)** Representative aldolase A protein expression images in knockdown HEp-2 cells. scRNA, scramble RNA transfected cells. ALDOA, aldolase A targeting siRNA transfected cells. **(B)** Representative images of inclusions formed in the knockdown HEp-2 cells. Chlamydiae infected knockdown cells at MOI 5, and were incubated for up to 3 days. Morphological changes in the inclusions were assessed under fluorescence microscopy at 3 (for *C. pneumoniae*) or 2 (for *C. trachomatis*) days after infection. Green, inclusions. **(C, D)** Changes in chlamydial infectious particle (IFU) numbers in the knockdown cells. Bacterial IFUs were evaluated by IFU assay. The IFU numbers were also assessed at the same time as above. Data show mean ± SD obtained from at least three independent experiments. *p < 0.05 versus value into scRNA-transfected cells. N.S., not significant. *C. pneumoniae* **(C)**. *C. trachomatis* **(D)**.

**Figure 4:**
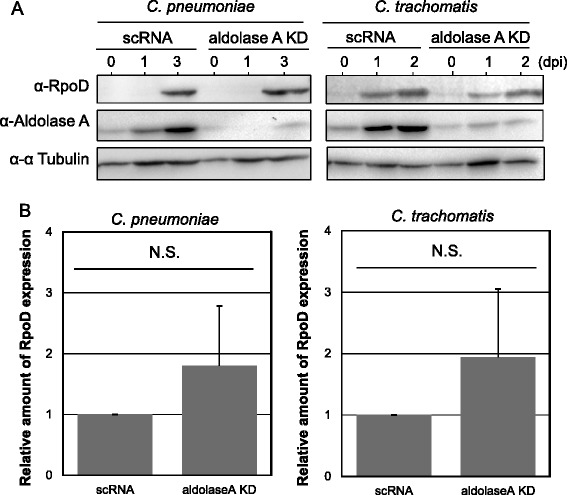
**Representative images showing chlamydial RpoD protein expression and its kinetic change into aldolase A knockdown cells. (A)** Representative images showing chlamydial RpoD protein expression in knockdown cells. Aldolase A knockdown cells infected with chlamydiae at MOI 5 were incubated for up to 3 days. RpoD protein expression was evaluated using Western blotting with a specific antibody against chlamydial RpoD. **(B)** Kinetic change of chlamydial RpoD protein expression in aldolase A knockdown cells at 3 (for *C. pneumoniae*) or 2 (for *C. trachomatis*) days after infection. Band-density values were evaluated by NIH Image J software. Data show mean ± SD obtained from at least three independent experiments. N.S., not significant.

Although it is not clear why chlamydiae should sequester host glycolytic enzymes such as aldolase A into infected cells, it is possible that glycolytic enzymes may be signaling molecules that sense viral or bacterial infection in the host cells, and that diminishing the signals may benefit chlamydial survival in the host cells. To confirm this hypothesis, we constructed transient aldolase A knockdown cells using siRNA, and assessed whether chlamydial growth could change in these cells. First, successful silencing of aldolase A was confirmed by western blotting with a specific antibody against aldolase A (Figure 3A, upper panel right). Chlamydial growth was carefully monitored in the knockdown cells by observation of inclusion morphology, bacterial count (EB numbers) with IFU assay, and western blotting for bacterial RpoD expression. In contrast to the immunofluorescence staining patterns of chlamydial inclusions (Figure 3B), the number of *C. pneumoniae* IFUs significantly increased in the knockdown cells (Figure 3C), and such a tendency to increase bacterial numbers was also seen in the *C. trachomatis*-infected knockdown cells, but with no significance (Figure 3D). Furthermore, changes in expression of RpoD, which is a core component of RNA polymerase and constitutively expressed in chlamydiae [34], were monitored using western blotting with a specific antibody against RpoD. Similar to IFU assessment data, the expression of RpoD tended to increase in the knockdown cells with either *C. pneumoniae* or *C. trachomatis* infection (Figure 4). We concluded that aldolase A silencing enhanced chlamydial growth. In addition, we confirmed by trypan blue staining that viability of aldolase A knockdown cells was maintained, indicating that the influence of dead cells was minimal in chlamydial growth (data not shown).

As mentioned above, the exact reason why sequestering aldolase A is required for stimulating chlamydial growth in infected HEp-2 cells remains undetermined. Recent work has shown that multifunctional glycolytic enzymes including aldolase A have so-called “moonlight functions” [35,36]. For example, it is well known that aldolase A binds to F-actin [37], and this binding step is important for secondary moonlight functions, such as vesicle trafficking [37], or cell motility [38]. Furthermore, aldolase A sequesters WASP and affects WASP/Arp2/3 complex associating with actin remodeling [39,40]. WASP/Scar proteins stimulated by Rho family small GTPases in turn activate the Arp2/3 complex to create new actin filaments [41]. More importantly, a recent study has indicated that caspase-1 digestome senses fragmented glycolytic enzymes, including aldolase A, digested in infected cells, resulting in cell death; a novel host defense system [42]. Based on these findings, we now speculate that sequestering aldolase A by CopN may be a survival strategy of chlamydiae in infected host cells, via preventing host cell death. Similar to *C. pneumoniae*, *C. trachomatis* growth was stimulated in aldolase A knockdown cells.

### Aldolase A gene expression tends to increase in *C. pneumoniae*-infected cells

**Figure 5:**
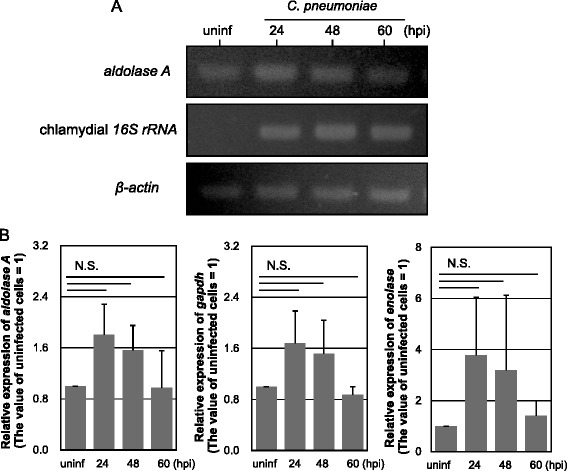
**Expression of genes encoding glycolytic enzymes including aldolase A, GAPDH and enolase, and the kinetic changes in HEp-2 cells with chlamydial infection. (A)** Representative RT-PCR images showing expression of genes encoding aldolase A, chlamydial 16S rRNA and β-actin in infected HEp-2 cells. *C. pneumoniae* infected cells at MOI 5 were collected at 24, 48 and 60 h. uninf, uninfected HEp-2 cells. **(B)** Kinetic changes in glycolytic enzyme gene expression in HEp-2 cells with chlamydial infection. The gene expression changes were evaluated by qRT-PCR. The values were normalized to that of *β-actin*. Data show mean ± SD from at least three independent experiments. N.S., not significant.

To understand the possible fate of aldolase A sequestered by CopN effector in *C. pneumoniae*-infected cells, the changes in expression of glycolytic enzyme genes including *aldolase A*, *gapdh* and *enolase* were monitored in parallel using qRT-PCR in infected and uninfected HEp-2 cells. Chlamydial infection to HEp-2 cells tended to enhance aldolase A gene expression as compared with the uninfected cells (Figure 5A). The slight increases in *gapdh* and *enolase* expression were also seen in the infected cells (Figure 5B, middle and right panels). Although further study is needed to clarify this, there may be a backup system to recover unexpected damage with abnormal metabolic pathways, occurring by sequestering glycolytic enzymes into host cells. It has been reported that changes in expression of glycolytic enzymes by bacterial infections have been shown in *Drosophila melanogaster* [43]. This finding presumably supports our hypothesis.

## Conclusions

This is believed to be the first study to show the interaction between *C. pneumoniae* T3SS effector CopN and human aldolase A. Although more experiments are needed to reveal why CopN should bind to aldolase A, sequestering aldolase A may be beneficial to bacterial growth in infected host cells, contributing to our understand of chlamydial pathogenesis and complicated host–parasite relationships.
